# Non-CpG methylation biases bisulphite PCR towards low or unmethylated mitochondrial DNA: recommendations for the field

**DOI:** 10.1093/eep/dvaa001

**Published:** 2020-02-04

**Authors:** Margaret J Morris, Luke B Hesson, Neil A Youngson

**Affiliations:** d1 School of Medical Sciences, UNSW Sydney, NSW 2052, Australia; d2 Prince of Wales Clinical School and Lowy Cancer Research Centre, UNSW Sydney, NSW 2052, Australia; d3 The Institute of Hepatology, Foundation for Liver Research, London, SE5 9NT, UK; d4 Faculty of Life Sciences and Medicine, King’s College London, London, UK

**Keywords:** non-CpG methylation, mtDNA methylation, bisulphite sequencing, mitochondrial DNA, PCR bias

## Abstract

Mitochondrial DNA (mtDNA) is a circular genome of 16 kb that is present in multiple copies in mitochondria. mtDNA codes for genes that contribute to mitochondrial structure and function. A long-standing question has asked whether mtDNA is epigenetically regulated similarly to the nuclear genome. Recently published data suggest that unlike the nuclear genome where CpG methylation is the norm, mtDNA is methylated predominantly at non-CpG cytosines. This raises important methodological considerations for future investigations. In particular, existing bisulphite PCR techniques may be unsuitable due to primers being biased towards amplification from unmethylated mtDNA. Here, we describe how this may have led to previous studies underestimating the level of mtDNA methylation and reiterate methodological strategies for its accurate assessment.

## Non-CpG Methylation Biases Bisulphite PCR towards Unmethylated Alleles with Standard Primer Design

The existence of mitochondrial DNA (mtDNA) methylation has been controversial for decades (reviewed in [1–3]). However, in the last 10 years there has been a growing consensus for its existence, and further, its functional relevance, including in environmental responses [4–12]. Three recent publications [13–15] are strong contributions to this shift in opinion as they provide technical advancements, as well as information on regional CpG and non-CpG mtDNA methylation, regulation by methyltransferases and associations with disease. The strength of evidence in these studies stems from innovative new techniques and inclusion of controls which are crucial for investigating this unique, small, circular genome of prokaryotic origin that exists within eukaryotic cells. The aim of this perspective is not to add to the number of recent reviews on mtDNA methylation but to highlight a technical problem which stems from discoveries in the three recent publications. This methodological issue has to our knowledge not previously been highlighted in relation to mtDNA methylation.

The most widely used technique for the study of mtDNA methylation is targeted/locus-specific/amplicon bisulphite sequencing [4–7, 9, 10, 16–19]. This technique allows identification of individual nucleotide methylation state within a chosen locus as an unmethylated cytosine is represented by a thymine following PCR, whereas a methylated cytosine is protected from bisulphite conversion and remains a cytosine. Targeted bisulphite sequencing was used in 11 of the 16 publications that investigated mtDNA methylation listed in Pubmed in the period between the 1 August 2018 and 28 October 2019. Several studies [8, 13, 15, 19] observed lower levels of mtDNA methylation with this technique than were detected in the same samples with whole-genome bisulphite sequencing (WGBS). WGBS utilizes bisulphite treatment followed by shotgun next-generation sequencing. Indeed, the observations of low DNA methylation levels with targeted bisulphite sequencing have even been used as evidence to support the idea that mtDNA methylation is at extremely low levels, or non-existent [7, 8, 16–18]. To explain this difference between targeted bisulphite sequencing and WGBS, Patil *et al.* [13] suggested that it could be due to PCR-based sequencing assays (pyrosequencing, methylation-specific PCR) usually only assaying methylation at CpG sites. Therefore, the high levels of methylation at non-CpG (CpA, CpT, CpC) sites is not detected [10, 13].

**Figure 1:**
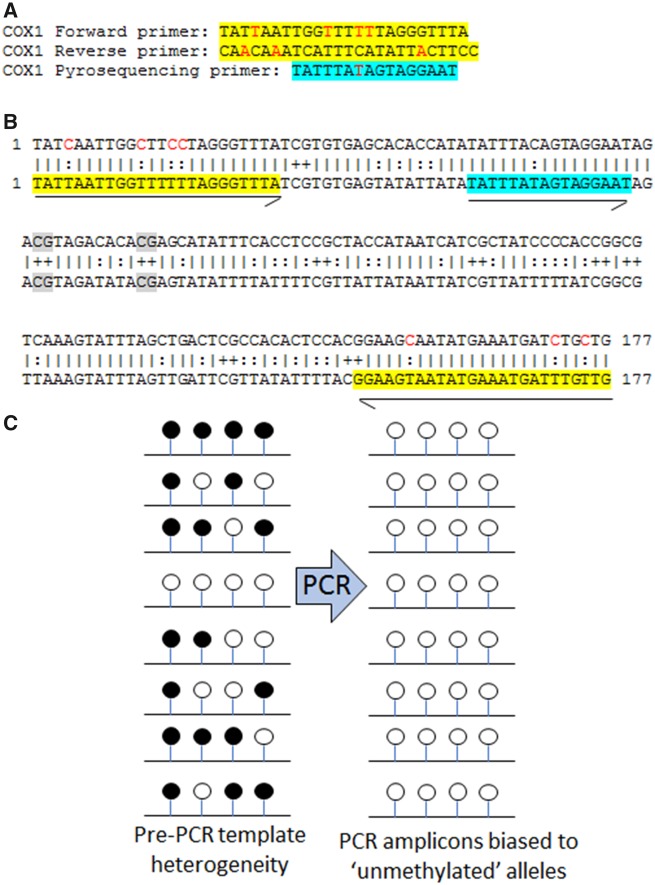
PCR from bisulphite-converted DNA in regions with non-CpG methylation is biased towards amplification of unmethylated alleles if the primer contains cytosines. (**A**) Typical primers used for bisulphite sequencing with bases that hybridize to converted non-CpG cytosines (red text). (**B**) Alignment of unconverted (top) and converted (bottom) sequences with non-CpG cytosines indicated with colons and CpGs with plus signs. Locations of PCR and pyrosequencing primers highlighted in yellow and blue, respectively, and indicated with arrows. Converted non-CpG cytosines in primers in red text. (**C**) Schematic showing selective amplification in PCR from unmethylated alleles in regions with a high frequency of non-CpG methylation. Methylated/unconverted and unmethylated/converted cytosines indicated with black and white lollipops, respectively

Another reason that may make targeted bisulphite sequencing a less accurate method for measuring methylation in mtDNA is the inherent bias of these assays when a DNA template has high levels of non-CpG methylation (Fig. 1). As DNA methylation in the mammalian nuclear genome is predominantly at CpG dinucleotides, current bisulphite PCR primer design methodologies [20] ensure that the primers contain thymines that correspond to converted non-CpG cytosines (CpA, CpC, CpT). These thymines are intended to promote amplification from fully converted templates. However, the assumption that all non-CpG cytosines are converted makes most targeted bisulphite sequencing primers unsuitable in regions that do in fact contain significant levels of non-CpG methylation [21]. In these regions, standard bisulphite sequencing primers will selectively amplify from the templates with the least methylation (Fig. 1C). For example, in the recent study by Patil *et al.*, the forward and reverse primers used to assay the MT-COX1 gene region in mtDNA contain four and three non-CpG cytosines, respectively (Fig. 1A and B), which would explain why they observed lower methylation in the bisulphite sequencing assay than with WGBS.

## Methodological Approaches to Compensate for the Presence of Non-CpG Methylation

Unlike most mammalian nuclear genomes, non-CpG methylation is abundant in plant genomes. Accordingly, plant researchers have proposed methods [22] to allow bisulphite sequencing in regions with non-CpG methylation that could be applied to mammalian mtDNA. These methodological changes include using primers that contain no cytosines or are degenerate in that they contain C/T at non-CpG cytosines (or G/A nucleotides on the complementary strand) so that 100% complementary primers are available for any potential combination of bases across the primer region. However, degenerate primers will have different proportions of cytosines and therefore differences in binding affinities and in optimal annealing temperatures. This means that higher PCR annealing temperatures are likely to ultimately report higher levels of methylation than lower annealing temperatures. Therefore, each primer pair must be tested with control templates [19, 22] of known percentages of C and T (or G and A) to identify the annealing temperature required to faithfully replicate the methylation level of the template.

A further risk of degenerate primers is that the introduced variation in primer sequence increases the possibility of non-specific amplification. If the mtDNA purification procedures do not adequately exclude the nuclear genome, degenerate primers increase the likelihood of amplifying mtDNA-like sequences from nuclear DNA [23]. One solution to this problem may be the use of sequencing technologies such as PacBio and Oxford Nanopore as the longer reads can be unambiguously identified as being from mtDNA [24].

## General Checklist for Best Practice Methodologies for Studying mtDNA Methylation

The unique challenges for analysing the epigenetic state of mtDNA have, throughout the development of the field, inspired innovative work to surmount those challenges. In Table 1, we list recommendations of the current best practices for DNA methylation analysis in mtDNA. For guidance purposes, we have only indicated a few recent studies that best demonstrate or describe these issues.

**Table 1:** best practices for assaying mtDNA methylation

All methodologies	
Confirm results with multiple techniques e.g. WGBS, MeDIP, mass spectrometry, PacBio, Nanopore	[13, 14, 19]
Enrich mitochondria or mtDNA prior to assaying to avoid unintentional detection of the nuclear genome	[13, 14, 19]
Present evidence of success of enrichment (e.g. qPCR of mtDNA vs nuclear DNA)	[13, 14]
Bisulphite methodologies	
Spike sample with control DNA for evaluation of conversion efficiency	[14, 19]
Linearize mtDNA prior to bisulphite conversion to avoid secondary structure effects	[13, 14, 16, 19, 25]
In WGBS separately analyse and present data from light and heavy strands	[13, 14]
Measure CpG and non-CpG methylation	[10, 13, 14]
Differentiate between 5mC and 5hmC	[14, 19, 26]
Use PCR primers or controls to account for non-CpG methylation	No examples of primers at present. Suitable positive and negative controls in [13, 19, 22]

In summary, given the rapidly increasing interest in the field of mtDNA methylation, more research is urgently required to investigate the underlying mechanisms and the functional role of methylation in development, environmental responses and disease. Targeted bisulphite sequencing has proven to be a quick and reliable method for assaying methylation levels in the nuclear genome. However, the discovery of widespread non-CpG methylation in mtDNA, means that adaptations to currently used methodologies are required to make targeted bisulphite sequencing a viable tool for researching this unique genome. Finally, as non-CpG methylation is present at significant levels in the nuclear genome in the brain and pluripotent cells [21], the approaches described here may also be adopted more broadly for the analysis of nuclear DNA.

*Conflict of interest statement*. None declared.
